# Cytoskeletal Proteins of *Actinobacteria*

**DOI:** 10.1155/2012/905832

**Published:** 2012-02-08

**Authors:** Michal Letek, María Fiuza, Almudena F. Villadangos, Luís M. Mateos, José A. Gil

**Affiliations:** Instituto de Biología Molecular, Genómica y Proteómica (INBIOMIC), Departamento de Biología Molecular, Área de Microbiología, Facultad de Biología, Universidad de León, 24071 León, Spain

## Abstract

Although bacteria are considered the simplest life forms, we are now slowly unraveling their cellular complexity. Surprisingly, not only do bacterial cells have a cytoskeleton but also the building blocks are not very different from the cytoskeleton that our own cells use to grow and divide. Nonetheless, despite important advances in our understanding of the basic physiology of certain bacterial models, little is known about *Actinobacteria*, an ancient group of Eubacteria. Here we review current knowledge on the cytoskeletal elements required for bacterial cell growth and cell division, focusing on actinobacterial genera such as *Mycobacterium, Corynebacterium*, and *Streptomyces*. These include some of the deadliest pathogens on earth but also some of the most prolific producers of antibiotics and antitumorals.

## 1. Introduction

All cells require cytoskeletal proteins for cell division and growth [1]. These structural components are essential for the maintenance of cell shape as well as for other dynamic processes critical for the cell, such as chromosomal segregation, the equal partitioning of cytosolic material, cell polarization, and motility [2].

The ubiquity of the cytoskeletal proteins reflects their early evolutionary acquisition and bacterial origin [3]. In fact, it is difficult to imagine an adaptable free-living cell without a versatile internal cytoskeleton. However, this notion is very recent since only just a decade ago it was thought that bacteria lacked a cytoskeleton. Instead, the required cell membrane support was assumed to be provided by the bacterial cell wall, which was thus considered to function as an “exoskeleton,” forming a physical barrier that contained the hydrostatic internal cell pressure and prevented the rupture of the cell membrane [4].

In fact, this exoskeleton does determine the characteristic shape of a bacterial cell, since in the absence of cell wall rod-shaped bacteria lose their morphology and become perfect spheres. But given that the chemical composition of the bacterial cell wall is essentially the same in the vast majority of Eubacteria (it is basically made of peptidoglycan or murein), it was also recognized that other factors must drive the determination of bacterial cell shape [5]. Osmotic pressure was thought to have some role in this process albeit a limited one, in view of the high morphological variability observed in different wild-type bacterial species and in strains carrying mutations in the different genes involved in cell morphology determination (morphogenes) [5]. In addition, and despite their apparent simplicity, bacterial cells undergo rapid and precise division, including chromosomal segregation and equal partitioning of the cytosolic contents, which would be impossible without the existence of an extremely dynamic but highly accurate internal organization [2, 5].

However, it is only now becoming clear that bacteria have an internal cytoskeleton made up of homologues to eukaryotic tubulin (FtsZ), actin (MreB/Mbl), and intermediate filaments (crescentin) [6]; there are even bacteria-specific cytoskeletal families of proteins, such as MinD [7] and bactofilins [8], without homologues in eukaryotes. All of these bacterial cytoskeletal proteins have pivotal roles on cell-wall synthesis and, consequently, cell-shape determination [7]. Here we review the most recent data regarding the bacterial cytoskeleton, with special emphasis on cell-shape determination by *Actinobacteria*, one of the three major bacterial groups.

*Actinobacteria* are gram positive with high GC-content genomes [9]. This ancient group of bacteria is one of the most interesting in terms of industrial and medical applications, but at the same time it is clearly understudied. Many actinobacterial species are industrially important for the bioconversion and production of antibiotics, antitumorals, amino acids, and vitamins [9]. There are also medically important species in this group, such as *Mycobacterium tuberculosis, Mycobacterium leprae*, and* Corynebacterium diphtheriae*, the causative agents of tuberculosis, leprosy, and diphtheria, respectively [9]. Yet, despite the enormous relevance for humans, little is known about the basic physiology of these and other actinobacterial species.

Progress in this field has been partially hampered by the lack of molecular tools, but mainly because of its complexity. The cell morphology of *Actinobacteria* varies enormously, from the almost coccoid cellular shape of *Rhodococcus* spp. to the fungal-like hyphae of *Streptomyces* spp. [10]. Furthermore, some of these bacteria have an outer lipidic layer composed of mycolic acids; this layer is essential for morphogenesis and for resistance to antimicrobials and to different stress conditions [11, 12]. In addition, some of these species sporulate, which requires two distinct molecular programs for cell-shape determination [13, 14]. Finally, recent data have demonstrated that *Actinobacteria* possess genus-specific morphogenes [15] that clearly differentiates them from each other but also further complicates the study of cytokinesis in this bacterial group.

## 2. Cytoskeletal Proteins Involved in Cell Division

Cell division in bacteria is governed by the tubulin-like protein FtsZ, a GTPase widely conserved and located within a cluster of genes involved in division and cell-wall synthesis (cluster *dcw*) [16]. During division, the cell membrane is constricted at the midcell, and peptidoglycan is synthesized to create a new cell wall in between the two newly formed daughter cells [17, 18]. This process starts with the polymerization of FtsZ and the assembly of the so-called Z-ring, a scaffold of cell division proteins that also generates the force needed for cell constriction [19]. No other protein is able to locate at the midcell during cell division before FtsZ; therefore, Z-ring assembly is the first step in bacterial cell division [20]. This structure recruits other proteins with different roles in cell division and cell-wall synthesis, creating a macromolecular complex called the divisome [20]. The composition of the divisome varies between different species, but a consensus has been established mainly based on *Escherichia coli *as the model organism.

There are positive and negative spatiotemporal regulators of FtsZ assembly that function to establish the exact location of cell division at the midcell. This results in two symmetrical daughter cells with an equal distribution of DNA and cytosolic material [17, 18]. The best studied FtsZ inhibitors are the MinCD and nucleoid occlusion systems. The MinCD system inhibits FtsZ polymerization at the cell poles to prevent asymmetrical division events [21, 22]. The nucleoid occlusion system, mediated by Noc/SlmA, is basically an inhibitor of FtsZ polymerization at those cellular locations where chromosomal DNA is present. This prohibits the unequal distribution of genetic material during cell division [22]. The combination of the two systems leaves the midcell after cell elongation and DNA replication as the only place available for FtsZ polymerization [17, 18].

Once the timing and location of cell division have been established, FtsZ polymerizes to generate the basic scaffold of the divisome, the Z-ring. The first proteins to be recruited to the Z-ring are FtsA and ZipA, which comprise the core of the divisome in *E. coli*. FtsA and ZipA simultaneously bind to FtsZ and the cell membrane, thus stabilizing the Z-ring [23, 24]. Once the two proteins are located at the midcell, the remaining proteins of the divisome are sequentially recruited [18, 20]: (1) the FtsEX complex, which may facilitate constriction [25]; (2) FtsK, which is required for chromosome segregation [26]; (3) FtsQLB, a bridge protein complex between the core of the divisome and the proteins involved in peptidoglycan synthesis [27, 28], such as (4) FtsW and FtsI, a peptidoglycan precursor translocator and a penicillin-binding protein (PBP), respectively [29, 30]; finally (5) FtsN and the amidases AmiA, AmiB, and AmiC, which mediate the peptidoglycan hydrolysis required for the final separation of the two newly formed daughter cells [31–34].

Several of the divisome proteins may have partially overlapping functions [35, 36], perhaps explaining why divisome composition is relatively variable in bacteria. In general, the positive (e.g., ZapA/B/C, ZipA, or SpoIIE) and negative (e.g., Noc/SlmA, MipZ, MciZ, SulA, EzrA, or MinCDE) spatiotemporal regulators of the Z-ring assembly are poorly conserved [16, 37–41]. In fact, actinobacterial genomes do not have homologues of these regulators [12, 42], and nucleoid occlusion has not been detected; that is, FtsZ polymerization may start over nonsegregated chromosomes [43, 44]. An exception to this rule is SepF, a conserved positive regulator of FtsZ assembly [45, 46]. However, SepF was dispensable for either the growth or the cell viability of *Corynebacteria *[47].

Conversely, actinobacterial-specific positive and negative regulators have been recently identified. The first Z-ring positive regulator in *Actinobacteria* was described using *M. tuberculosis* as a model [48]. FtsW acts as a translocator of peptidoglycan precursors through the cell membrane during cell division [29]. This protein was also found to be a direct interaction partner of FtsZ in *M. tuberculosis*, suggesting that FtsW anchors the Z-ring to the cell membrane. Consequently, FtsW could be involved in the positive regulation or stabilization of the Z-ring in *Actinobacteria*, which lack homologues of FtsA and ZipA [48–50]. In fact, a similar role has been proposed for FtsW in *Streptomyces coelicolor *but only during sporulation septation [51]. Also, the *Streptomyces*-specific SsgA and SsgB proteins have been described as positive regulators of FtsZ assembly during spore formation [52, 53]. In general, the sporulation mechanism of *S. coelicolor* is better studied than its vegetative cell division process. Finally, there is evidence that the FtsZ-interacting protein A (FipA) from *M. tuberculosis* is a positive effector of cell division under oxidative stress conditions [54].

On the other side of the coin, there are also actinobacterial-specific inhibitors of FtsZ assembly. DivS is a cell-division suppressor that acts in response to DNA damage in *Corynebacterium glutamicum* [55]. PldP is a ParA-like protein that may be involved in the cell-division site selection of *C. glutamicum* [56]; a PldP null mutation generates minicells, a phenotype caused by the formation of septa at the cell poles and the generation of asymmetrical daughter cells with an unequal distribution of chromosomal DNA. ClpX directly interacts with FtsZ in *M. tuberculosis* and blocks its polymerization in response to various stress conditions, such as intramacrophage growth and antibiotic treatment [57]. Finally, the product of *crgA*, a small gene widely conserved in *Actinobacteria*, has been described as an inhibitor of *Streptomyces* cell division [58]. However, CrgA has been recently characterized as a facilitator of FtsI localization in *M. tuberculosis* [59], proving once again the complexity and variability of actinobacterial cell division.

## 3. Cytoskeletal Proteins Involved in Cell Elongation

In many bacillary bacteria, MreB actin-like homologues are essential for cell-wall elongation [60, 61]. The *mreB* gene is usually localized in the *mre* operon, together with *mreC* and *mreD* [62, 63]. The *mreBCD* cluster was identified based on the coccoid cell shape resulting from the mutation of these genes [62, 63]. MreB is an ATPase capable of polymerizing into long filaments in the presence of ATP or GTP [64–66]. During the last decade, it has been assumed that MreB forms helicoidal protofilaments that extend from pole to pole in the cell directing the synthesis of the lateral cell wall and cell elongation in many rod-shaped bacteria [61, 67, 68]. However, recent evidence suggests that MreB localizes in discrete patches that move along the cell in the company of proteins involved in peptidoglycan synthesis and translocation of cell wall precursors: Pbps and RodA, respectively [69, 70]. MreCD and also RodZ, a conserved membrane protein, are thought to act as a link between MreB and the peptidoglycan synthesis machinery [69–73]. In this new model, old peptidoglycan strands act as scaffolds of new cell wall synthesis, and the movement of the molecular machines involved in this process is powered by peptidoglycan polymerization [69, 70, 74]. MreB filaments could be required for controlling the orientation and movement of these molecular complexes and/or the recruitment of peptidoglycan precursors for their translocation across the membrane [70].

Nonetheless, MreB is essential for maintaining the cell wall synthesis and cell elongation in *Bacillus subtilis, E. coli *or* Caulobacter crescentus *[61]. In all these bacterial models the incorporation of new cell wall material occurs at the midcell during cell division (sustained by FtsZ) and at the lateral walls during cell elongation in an MreB-dependent fashion, while the polar ends of the cell are inert [68, 75, 76]. In microorganisms with a coccoidal shape and devoid of *mreBCD* genes like *Streptococcus pneumoniae*, cell wall synthesis during either cell division or cell elongation is only accomplished at the division site [68].

In contrast, all *Actinobacteria* studied thus far grow apically, that is, by the insertion of new peptidoglycan at the cell poles rather than at the lateral wall, which is inert [77–79]. This growth is independent of MreB; in fact, all mycobacterial and corynebacterial genomes sequenced to date lack *mreB* homologues, whereas *Streptomyces* use MreB homologues only for sporulation [80–82]. This form of cell elongation is sustained by the protein DivIVA, which localizes at the cell poles and is essential for cell viability [77–79, 83]. DivIVA polymerizes at the cell poles to generate an internal cytoskeleton that supports and recruits the cell-wall synthesis machinery in *Actinobacteria*. DivIVA also localizes at the division site, suggesting its role in the maturation of the newly formed cell poles [78].

In *Actinobacteria*, a change in DivIVA protein levels leads to strong morphological alterations. Specifically, the low-level expression of DivIVA produces coccoidal cells in the rod-shaped *Corynebacterium* and *Mycobacterium*; this is because the lack of DivIVA abolishes the polar synthesis of peptidoglycan [78, 79]. Presumably, cell-wall synthesis occurs uniformly along the cell, creating perfect spheres; alternatively, the cell-division apparatus is able to create a cell wall that is sufficient to allow cell enlargement [78, 79]. This latter hypothesis is more plausible since the only place where peptidoglycan synthesis has been clearly detected in DivIVA-depleted cells is the division septum. Interestingly, DivIVA is essential in *Actinobacteria*; that is, a knockout mutant is lethal, suggesting that this protein has another, yet unexplored role apart from the internal support of polar cell-wall synthesis [77, 78].

The overexpression of DivIVA creates large asymmetrical cells that are enlarged at one polar end, where the majority of the protein localizes [77–79, 83, 84]. DivIVA was shown to oligomerize through two coiled-coil regions [85, 86] whereas the highly conserved N-terminal domain is probably required for the protein's initial localization to the membrane at the polar end of the cell [87]. In *Actinobacteria*, all three domains are essential for DivIVA function; however, once DivIVA is localized at the cell's new polar end, self-interaction is probably the main force required for the protein's localization [88–90]. Overexpression of DivIVA results in greater amounts of the protein at the cell poles, which in turn become very active sites of peptidoglycan synthesis [78]. Presumably, after cell division the old polar end will have initially a larger amount of DivIVA than the recently created polar end. Therefore, the old polar end will attract a larger amount of DivIVA, which eventually will lead to an asymmetrical club-shaped cell. The balance of DivIVA levels radically changes not only the length of the cell but also its diameter [77–79, 83].

**Figure 1:**
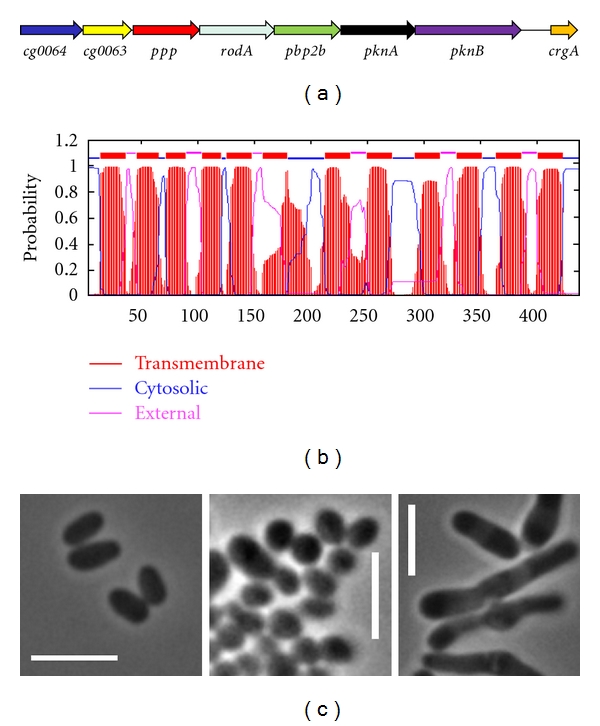
RodA is required for rod-shape maintenance of *C. glutamicum*. (a) In *Actinobacteria*, *rodA* is located within the conserved *pkn* cluster. (b) Prediction of transmembrane helices of RodA using TMHMM 2.0 software [93]. (c) Partial depletion of RodA generates coccoid cells (central) in the rod-shaped *C. glutamicum* (left), whereas a RodA overexpression results in club-shaped cells (right). M. Fiuza, unpublished results.

All three DivIVA domains can be exchanged without drastic consequences [88]. In fact, the only conserved region of this protein is the N-terminal domain whereas the coiled-coil regions differ greatly in size and sequence even between highly related species [91]. However, DivIVA proteins from *B. subtillis* or *S. pneumoniae* are not able to complement the lack of DivIVA in *C. glutamicum*, in contrast to DivIVA proteins from other *Actinobacteria *[78]. This suggests that actinobacterial DivIVA proteins have an unknown signature or motif in their sequences that enables their role in polar cell-wall synthesis. This is probably mediated by protein-protein interactions with class B high-molecular-weight PBPs, directly involved in cell-wall synthesis, and RodA, an essential membrane protein probably involved in the transport of peptidoglycan precursors outside the cell during cell growth [92]. RodA is in fact required for rod cell shape determination in *C. glutamicum* (Figure 1).

## 4. Other Cytoskeletal Proteins

In bacteria, tubulin-like proteins are required for cell division, whereas actin-like proteins maintain cell elongation, with the notable exception of *Actinobacteria*, in which, as discussed above, DivIVA directs polar cell-wall extension. Coiled-coil rich proteins such as DivIVA are common elements of the cytoskeleton of all organisms, no doubt due to their ability to oligomerize through self-interaction [94, 95]. In eukaryotes, intermediate filaments (IF) are the best example of cytoskeletal coiled-coil proteins, and in bacteria, IF-like proteins were recently identified.

The first bacterial IF-like protein was crescentin [96], which was described in *C. crescentus*. In this bacillary bacterium, mutations in the gene encoding crescentin, *creS*, straighten the curved shape of the cells. Crescentin participates in the formation of helicoidal and filamentous structures along the cell. The protein redirects cell-wall synthesis controlled by MreB to ensure a curved bacillus instead of a straight rod [96].

The main characteristics of IF-like proteins are their lack of enzymatic activity and their capacity for *in vitro* polymerization in the absence of any cofactors [97]. Coiled-coil self-interaction is highly resistant to mechanical stress [94, 95], making IF-like proteins perfect candidates for cytoskeletal proteins. However, the sequence homology between IFs and crescentin is very low, although all these proteins have in common a high content of coiled-coil regions [96]. Since many different amino acid sequences are able to adopt the coiled-coil structure, sequence conservation is not essential, which makes it very difficult to identify IF-like proteins in bacteria strictly by homology. Despite the low level of sequence conservation, coiled-coil regions consist of a repeated pattern of seven amino acids in which the first and fourth positions are always hydrophobic. This pattern allowed the identification of other bacterial IF-like proteins besides crescentin by *in silico* mining of coiled-coil regions [15, 98]. It is now becoming clear that IF-like proteins are amply distributed in bacteria; however, a cytoskeletal role has been attributed only to a few of them: CfpA in spirochaetes [99], CcrP in *Helicobacter pylori *[100], FilP in *S. coelicolor* [98], and RsmP in *C. glutamicum *[15].

CfpA is found exclusively in spirochaetes, where the protein forms helicoidal filamentous structures along the cell that are required for cell division and chromosomal segregation [99]. In *H. pylori*, CcrP also forms filamentous structures both *in vitro* and *in vivo*, but the protein seems to be required in the maintenance of this bacterium's helicoidal shape and its motility [100, 101]. CcrP proteins vary enormously in sequence between different strains of *H. pylori*; this variability may be linked to the high morphological differences encountered in clinical isolates [100].

FilP is an IF-like element of *S. coelicolor *[98]. Mutations in the *Streptomyces filP* gene cause strong morphological alterations in this bacterium and a marked deficiency in cell growth. FilP is probably required for additional support during polar cell-wall synthesis in *S. coelicolor* and thus contributes to the mechanical resistance of hyphae [98].

RsmP has been identified only in *Corynebacteria *[15]. This IF-like element is overexpressed in response to the partial depletion of DivIVA. Similarly to *divIVA*, *rsmP* is an essential gene in *C. glutamicum*, and its partial inhibition has been shown to result in the formation of coccoid cells while its overexpression induces a club-shaped morphology [15]. RsmP is able to produce filamentous structures *in vitro* and *in vivo* along the cell. The most interesting feature of RsmP is the change of its subcellular localization depending on its phosphorylation state. RsmP is phosphorylated at three different residues by the serine/threonine kinases PknA and PknL (see below and [15]). The cellular localization of an RsmP phosphoablative mutant is indistinguishable from the native RsmP, it still forms long filamentous structures along the cell. In contrast, a phosphomimetic mutant localizes only at the cell poles of *C. glutamicum*, suggesting that the phosphorylation state of RsmP is involved in the modulation of polar peptidoglycan synthesis in *Corynebacteria *[15]. The discovery of RsmP demonstrated that *Corynebacteria* have a specific molecular system for establishing their rod-shape morphology, thus distinguishing them from other *Actinobacteria* such as *Mycobacterium* and *Streptomyces*.

It is worth mentioning that there is also an alternative to IF-like elements, specifically in Eubacteria. Bactofilins have been recently identified in *C. crescentus* as a bacteria-specific cytoskeletal family of proteins that provide structural support for peptidoglycan synthesis [8]. Bactofilins also polymerize in the absence of any cofactors, forming rod-shaped filaments *in vitro* and polar structures *in vivo* during stalk morphogenesis. These proteins are widely conserved in Eubacteria (with notable exceptions like *Actinobacteria*), suggesting a high structural and functional versatility [8].

## 5. Cell Shape Control by Phosphorylation of the Bacterial Cytoskeleton

A seminal report published in 2005 demonstrated for the first time that the bacterial cytoskeleton is controlled by eukaryotic-like serine/threonine phosphorylation [102]. In that paper, Kang et al. definitively showed that two protein kinases, PknA and PknB, modulate cell shape in *M. tuberculosis* by changing the phosphorylation state of DivIVA [102]. Not much later, FtsZ was identified as another substrate of Pkn phosphorylation, thus linking the control of cell division with cell growth in *Actinobacteria* by a unique signal transduction system [103].

Both PknA and PknB are located within a highly conserved cluster in *Actinobacteria *[104]. This cluster includes: (1) two genes of unknown function with forkhead-associated (FHA) domains, (2) a phosphatase that antagonizes *pkn* kinases, (3) *rodA* and a *pbp* required for cell-wall synthesis during elongation, (4) *pknAB,* and (5) *crgA* (Figure 1(a)). Further work demonstrated that most of these genes are somehow related to cell-shape determination in *Actinobacteria *[59, 105–108].

Despite the high degree of conservation within *pkn* clusters, their functions have proven to be quite dissimilar when compared in different *Actinobacteria *[105]. The main differences in *pkn* regulation of actinobacterial cytokinesis probably involve DivIVA, the major coordinator of cell growth in these bacteria. In *M. tuberculosis*, PknA phosphorylates DivIVA, thus controlling cell-wall elongation [109]. In *C. glutamicum*, DivIVA seems to be not phosphorylated by PknA; instead, this kinase phosphorylates the *Corynebacterium*-specific RsmP protein, which in turn controls cell growth [15].

These distinctions are complicated by the possibility that Pkn kinases are directly involved in the modulation of the synthesis of peptidoglycan precursors, since the MurC and MurD peptidoglycan ligases are also phosphorylated by PknAB in *C. glutamicum* and *M. tuberculosis*, respectively [110, 111]. However, the biological significance of these observations has yet to be fully determined.

**Figure 2:**
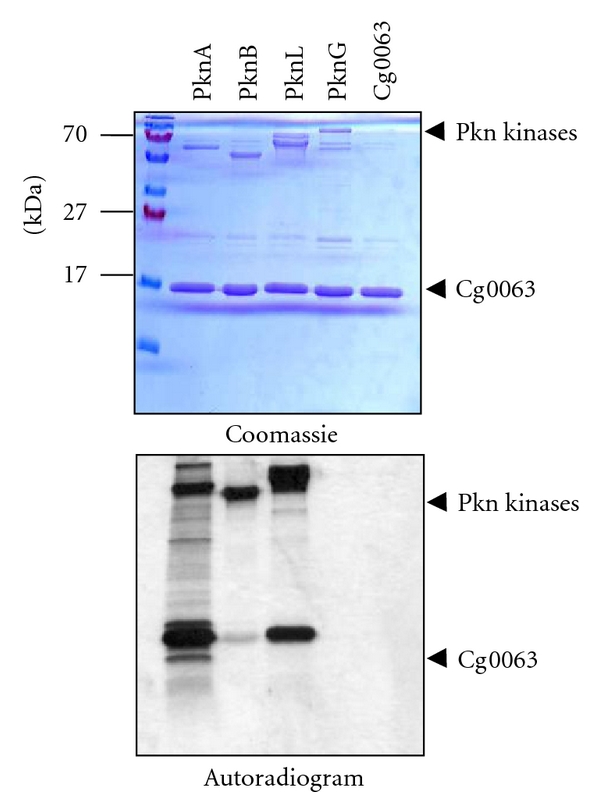
Cg0063 is phosphorylated *in vitro* by PknA and PknL in *C. glutamicum*. All four Ser/Thr Pkn kinases from *C. glutamicum* (PknA/B/L/G) and Cg0063 have been expressed and purified as described previously [105]. Then, Cg0063 was incubated alone or with the different Pkn kinases in the presence of [**γ**-^33^P] ATP for 30 min. Samples were separated by SDS-PAGE electrophoresis and stained with Coomassie Blue (upper panel) or visualized by autoradiography (lower panel). PknA/B/L kinases exhibit an autophosphorylation activity, whereas Cg0063 is mostly phosphorylated by PknA and PknL. M. Fiuza, unpublished results.

Finally, the *pkn* cluster contains two genes with FHA domains [*cg0064 (fhaA) *and* cg0063 (fhaB)* in *C. glutamicum*], a feature that will no doubt add another layer of complexity to our attempts to understand Pkn regulation of actinobacterial cell shape. FHA domains are phosphopeptide recognition motifs that specifically recognize phosphothreonine-containing epitopes for protein-protein interaction. In *C. glutamicum*, we determined that the Cg0063 (FhaB) protein is phosphorylated by PknA and PknL, another serine-threonine kinase located elsewhere in the chromosome (Figure 2). This indicates a possible role for the Cg0063 (FhaB) protein in cell-shape determination. The functions of other FhaAB proteins have been analyzed in *Actinobacteria* and include the maintenance of hyphal morphology in *S. coelicolor* [112] and the virulence of *M. tuberculosis* [113].

Further experimental work is needed before the *pkn* cluster is thoroughly understood; nevertheless, the evidence obtained to date strongly favors the conclusion that Pkn kinases direct actinobacterial cell division and cell elongation. Accordingly, PknA and PknB kinases have been identified as very promising targets for the development of new antituberculosis drugs [114, 115].

## 6. Final Remarks

During the last two decades, a good deal of progress has been made in our understanding of the basic physiology of bacteria. Once believed to be simple organisms with a low level of organization and totally unrelated to eukaryotes, bacteria are now recognized as very sophisticated forms of life that share most of the molecular tools used by our own cells to grow and replicate [1, 3, 7]. Actin-, tubulin- and IF-like proteins are being slowly identified and characterized in bacteria. Even bacterial-specific families of cytoskeletal proteins have been recently discovered [7, 8]. Most of the genes in the over-1000 bacterial genomes sequenced to date are still of unknown function; therefore many surprises probably await us. The high variability of cellular shapes in bacteria and their fantastic versatility make the study of prokaryotic cytokinesis very exciting but also extremely challenging [5]. The discovery of genus-specific molecular strategies guiding bacterial cell-shape determination equips us with unique targets for the development of new antimicrobial drugs, one of the main goals of the study of bacterial morphogenesis [116]. However, much more work is needed to completely unravel at least one model of bacterial cell division and cell growth. Yet this field of research has already yielded numerous practical applications, in the form of novel compounds that specifically inhibit FtsZ, MreB, or PknAB [114, 115, 117–119]. This progress could be vital to combating the inexorable development of new multi-drug-resistant pathogens appearing all around the world [120, 121].
